# Effects of the computer-assisted rehabilitation environment on patient rehabilitation: a systematic review and meta-analysis

**DOI:** 10.3389/fmed.2025.1733273

**Published:** 2025-12-05

**Authors:** Zhengbo Liang, Qing Huang, Dongmei He, Jing Wang, Ruixin Peng, Jing Zhang, Xiao Xiao

**Affiliations:** 1 Department of Anesthesia Surgery Center, Deyang People's Hospital, Deyang, China; 2 Department of Rehabilitation Medicine, Deyang People's Hospital, Deyang, China

**Keywords:** computer-assisted rehabilitation environment, recovery, neurology, musculoskeletal, meta-analysis

## Abstract

**Background:**

Computer-assisted rehabilitation environment (CAREN) integrates virtual reality, motion capture systems, and real-time feedback mechanisms to enhance patient rehabilitation results. This study aimed to determine the potential benefit of CAREN in increasing balance, cognition, and mental health in patients with neurological and musculoskeletal disorders.

**Methods:**

Systematic searches were carried out across PubMed, Embase, Cochrane Library, China National Knowledge Infrastructure (CNKI), and Web of Science databases for relevant studies published up to November 2025. The review comprised randomized, non-randomized controlled trials and single-arm studies evaluating the rehabilitative outcomes of CAREN treatment compared with standard therapy or no intervention in patients. The primary outcomes comprised balance function, cognitive functions, and mental health status; the secondary outcome was the incidence of adverse events. The quality of studies included in the review was evaluated using the Newcastle–Ottawa Scale, and the pooled effect size was computed using standardized mean differences (SMDs) or mean differences (MDs) for continuous outcomes. Statistical analysis was performed using Stata 18.0.

**Results:**

The database search yielded 3,553 records, of which 15 studies were included, and 9 had sufficient data for meta-analysis. CAREN training significantly improved balance, as indicated by higher Berg Balance Scale scores (SMD = 1.12; 95% CI = 0.08 to 2.16; *p* = 0.03; *I*^2^ = 92.64%), and cognitive function, as shown by increased Montreal Cognitive Assessment scores (MD = 0.44; 95% CI = 0.11 to 0.76; *p* = 0.01; *I*^2^ = 0.00%). Changes in fear of falling, assessed with the Falls Efficacy Scale-International (MD = −0.05; 95% CI = −0.36 to 0.27; *p* = 0.76; *I*^2^ = 0.00%), and depression, evaluated with the Beck Depression Inventory or Hamilton Depression Rating Scale (MD = −0.20; 95% CI = −0.62 to 0.22; *p* = 0.35; *I*^2^ = 70.64%), were non-significant. Additionally, adverse events were rare, with no serious cases reported.

**Conclusion:**

CAREN training appears to improve balance and cognitive function, while its effects on mental health are relatively limited. However, these findings should be interpreted with caution.

**Systematic review registration:**

PROSPERO, identifier (CRD420251172390).

## Introduction

Rehabilitation is an inherently multidimensional process, encompassing not only the restoration of motor functions but also the recovery of cognitive and emotional capacities (1, 2). Patients with neurologic disorders, such as Parkinson’s disease (PD), traumatic brain injury (TBI), cerebellar ataxia, multiple sclerosis (MS), and stroke, experience difficulties with balance, gait, and emotional regulation (3, 4). Similarly, patients with musculoskeletal disorders, such as transtibial amputation and osteoarthritis, experience comparable difficulties (5). These interrelated deficits compromise postural stability, mobility, independence, and overall quality of life. In addition, cognitive and emotional impairments can exacerbate motor control problems by interfering with attention, executive functions, and movement planning, creating a self-propelling cycle of disablement (6). Therefore, there is evidence that holistic approaches to recovery, addressing motor function, cognition, and emotional regulation, are necessary for achieving complete recovery in patients with neurological or musculoskeletal disorders.

Over the past two decades, virtual reality (VR)-based rehabilitation systems, which combine physical training with sensory interaction and are hypothesized to enhance cognitive engagement and affective motivation, have been introduced as powerful tools for promoting functional recovery and psychosocial outcomes (7). VR environments provide simulations that mimic real-life situations in a safer environment, such as obstacle courses and balance disruptions, to minimize risk during the learning process (8). Additionally, virtual reality has shown promising results in motor function recovery and improving balance and gait function during neuromuscular rehabilitation (9, 10). The interactive learning process in virtual reality ensures that there is multisensory integration (11, 12).

The present review specifically focuses on the computer-assisted rehabilitation environment (CAREN), an immersive VR-based platform that integrates full-motion analysis, dynamic virtual environments, and real-time feedback to achieve a multisensory rehabilitation program (13). CAREN was designed to overcome the limitations of conventional VR systems—particularly non-immersive or fixed-base platforms with static interfaces—by enabling realistic body–environment interaction through integrated biomechanical feedback and dynamic motion control (14). Through the integration of a multi-axis motion platform with three-dimensional (3D) visual displays, the patient can interactively experience dynamic environments that simulate real-world demands, therefore encouraging motor learning and balance control (15). The dynamic environments, such as obstacle courses or model ships, assess balance and locomotor functions, while simultaneous cognitive tasks target executive function, space orientation, and decision-making skills (16). Integrating these motor, cognitive, and emotional stimuli may potentially lead to neuroplastic adaptation and functional recovery, with emphasis on somatic as well as psychological gain (17, 18).

Although numerous studies have explored the rehabilitative potential of the CAREN system, the results remain inconsistent. Existing reviews have often focused on specific patient populations or isolated outcomes, leaving a gap in understanding the overall rehabilitative impact of CAREN. This meta-analysis aimed to systematically review current clinical trials and quantify the effects of the CAREN system on balance, cognition, and mental health in patients with neurological and musculoskeletal disorders, providing a more comprehensive assessment across diverse patient groups and outcome measures.

## Methods

This systematic assessment was performed adhering to the process indicated in the Cochrane Handbook for Systematic Reviews and illustrated according to the statements in the Preferred Reporting Items for Systematic Reviews and Meta-Analyses (PRISMA). The review protocol was registered in PROSPERO (CRD420251172390).

## Literature search

We conducted a systematic search of PubMed, Embase, Cochrane Library, China National Knowledge Infrastructure (CNKI), and Web of Science using an extensive list of keywords, including “CAREN,” “computer-assisted rehabilitation environment,” “virtual reality,” “computer-assisted rehabilitation,” “robot-assisted rehabilitation,” “immersive rehabilitation,” “rehabilitation,” “neurorehabilitation,” and “motor recovery,” to identify relevant studies. The search was limited to peer-reviewed journal articles published in English and Chinese up to November 2025. Two independent reviewers conducted the screening, with disagreements resolved by a third reviewer. To maximize reproducibility and facilitate further assessment, the detailed search strategy for each database is provided in Supplementary Table S1.

## Inclusion and exclusion criteria

Two independent researchers conducted the initial screening of titles and abstracts to identify studies that align with our predefined inclusion criteria: (1) patients requiring rehabilitation for neurological or musculoskeletal disorders and (2) studies involving CAREN, alone or in combination with other interventions, with pre- and post-outcome data, including randomized controlled trials (RCTs) and non-RCTs with conventional or no-intervention comparators. Given that the CAREN system is a novel treatment method, its exploratory evaluation is crucial, and single-arm designs provide valuable preliminary data to support future research, so we included them in this study; (3) studies that report rehabilitation outcomes, such as motor function recovery and cognitive function improvement, with a sample size of 10 or more patients; and (4) full-text articles published in English or Chinese. The exclusion criteria were as follows: (1) conference abstracts, case reports, letters, editorials, or review articles; (2) studies involving only healthy individuals; (3) studies with incomplete data, those that do not report relevant rehabilitation outcomes, or those that solely compare the effectiveness of CAREN training without assessing post-intervention improvements; and (4) duplicates or overlapping datasets from the same study population.

### Data extraction

The process that was followed in data collection entailed independent and duplicate data extraction using a standardized form. In that process, before data extraction took place, the two independent reviewers participated in a calibration process to ensure that there was homogeneity in study selection. In case there was no concordance after the evaluation, then the results would be adjudicated by another reviewer. Data items included the first author, year of publication, country of origin, duration of the study, design, sample size, participant demographics (age and gender), disease type, details concerning the CAREN training (e.g., number, duration, and frequency of sessions), results (primary outcomes included the Berg Balance Scale (BBS) for balance, the Montreal Cognitive Assessment (MoCA) for cognition, the Falls Efficacy Scale-International (FES-I), and the Hamilton Rating Scale for Depression (HAM-D) for mental health; the secondary outcome was safety), instruments for evaluation, and the virtual reality scenario and tasks utilized during the intervention. Corresponding authors were contacted for clarification or to obtain missing information when necessary.

### Quality assessment

Given that our review included RCTs, non-RCTs, and single-arm studies, we chose the Newcastle–Ottawa Scale (NOS) for bias assessment, as it provides a consistent framework for evaluating study quality across all designs. Bias potential was assessed independently by two reviewers using the NOS, with any disagreement resolved by consensus. The NOS considers three main dimensions: the quality of the sample, the control group, and representativeness; the adjustment for important confounders such as age, gender, and baseline status; and the appropriateness for the outcome measure, follow-up duration, and definability for the assessment. The system provides a star rating system, with a maximum of nine stars, where higher scores indicate higher quality studies (19).

### Assessing the certainty of evidence

The Grading of Recommendations Assessment, Development and Evaluation (GRADE) approach was used to evaluate the certainty of evidence with regard to each critical outcome. The certainty of evidence was rated to be high, moderate, low, or very low depending on the GRADE criteria with regard to domains such as study design, risk of bias, inconsistency, indirectness of evidence, imprecision, and publication bias.

### Statistical analysis

Data analysis was performed using Stata 18.0 software. Standardized mean differences (SMDs) or mean differences (MDs) were used as effect sizes to investigate the changes in rehabilitation outcomes pre- and post-intervention, along with their corresponding 95% confidence intervals (CIs). The primary outcomes were balance, cognition, and mental health, while the secondary outcomes were adverse events. Meta-analyses were performed when at least three studies used comparable instruments for a given outcome. The heterogeneity of the results from the meta-analysis was assessed through the *I*^2^ statistic. Heterogeneity was classified as low (< 25%), moderate (25 to 50%), or considerable (≥ 50%). A random-effects model was used when significant heterogeneity was present, while a fixed-effects model was applied when heterogeneity was not significant. In addition, publication bias was assessed using funnel plots and Egger’s test to confirm the validity and reliability of the results. Statistical significance was defined as a *p*-value <0.05.

## Results

### Literature search

**Figure 1:**
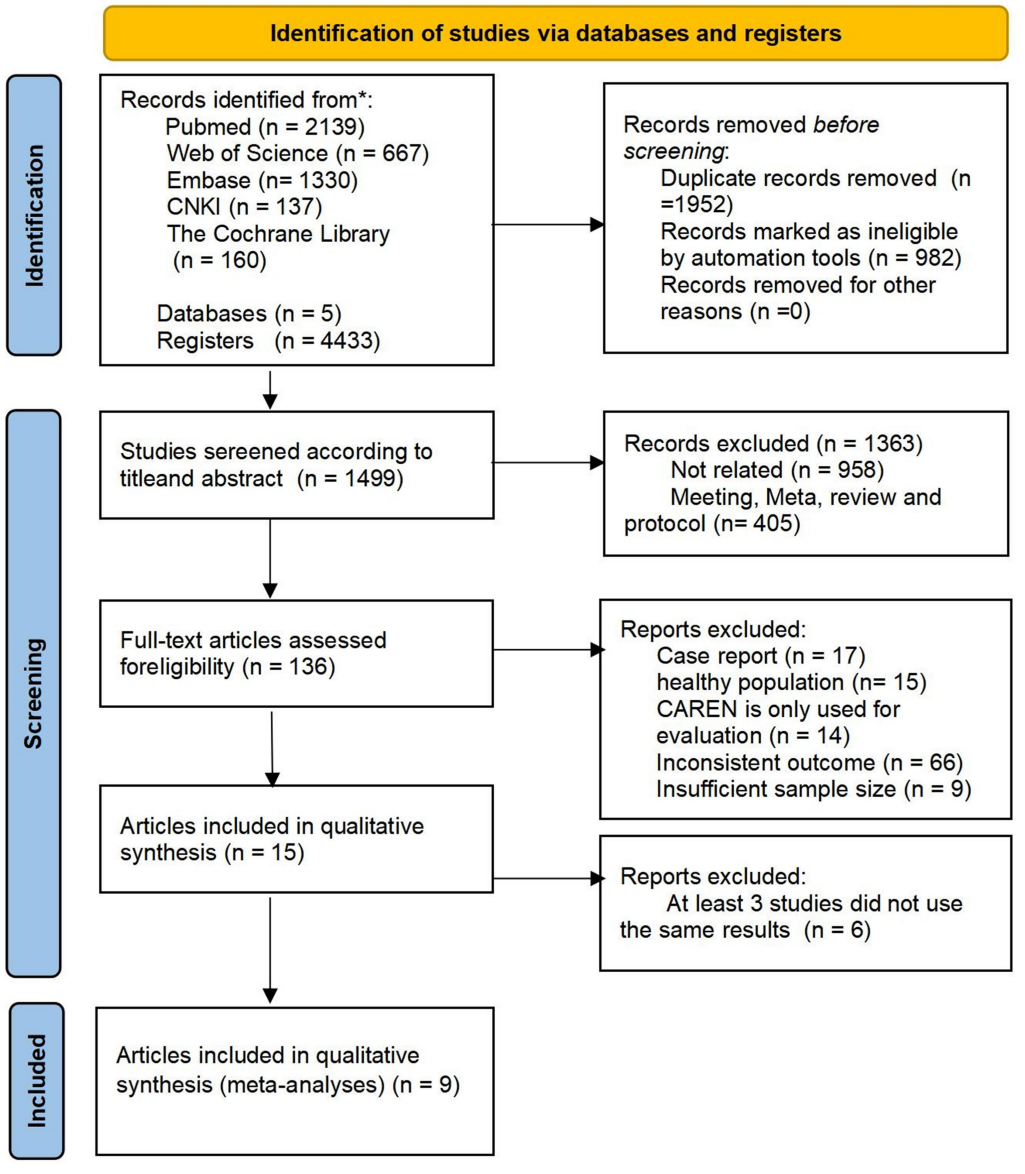
Flow diagram of study selection.

Screening systematic literature searching on PubMed, Web of Science, Cochrane Library, CNKI, and Embase yielded 4,433 records. The ineligible records and duplicates were automatically filtered through programming, and 1,499 studies were left to be included. Title and abstract screening further led to the selection of 136 papers for full-text reviewing. A total of 136 full-text articles were then assessed for eligibility, resulting in the inclusion of 15 studies for the qualitative synthesis (20–34) and 9 for the meta-analysis (23, 24, 27, 28, 30–34) (Figure 1). The inter-rater agreement was substantial (*κ* = 0.82) between the reviewers during full-text screening.

### Characteristics of credible studies

As shown in Table 1, the sample sizes of the included studies were not homogeneous, with 10 to 214 participants, for a total sample of 808 persons, the majority of whom were male. The studies took place in various locations, the majority coming from the USA (5 studies), followed by Italy (4 studies), the Netherlands (3 studies), China (2 studies), and Israel (1 study). The participants were mostly those with the following diseases: multiple sclerosis (MS), traumatic brain injury (TBI), Parkinson’s disease (PD), and traumatic transtibial amputation (TTA). One study included children (with cerebral palsy), and the remaining studies included adult participants. Control interventions were diverse, comprising no intervention, conventional physiotherapy/usual care, and alternative CAREN exposures, namely different populations performing identical CAREN tasks or the same population receiving lower-frequency CAREN (Supplementary Table S2). Among the studies, the training sessions with the CAREN were variable in terms of duration, ranging from 1 to 12 weeks, and the prevailing training frequency was twice a week. Seven studies assessed balance (24, 25, 27–29, 32, 33), eight studies assessed gait parameters (20–23, 26, 28, 29, 32), four studies assessed cognitive performance (23, 30, 32, 34), and nine studies assessed mental health (24, 25, 28–34).

**Table 1:** Summary table of included studies.

ID	Country	Study period	Study design	Sample size	Age	Gender (M/F)	Disease type	Sessions	Outcome
Gates, 2012 (20)	USA	2011–2012	Non-RCT	34	25–50	7/27	TTA	15 sessions, 5 weeks, 3 times a week	Gait parameters and safety
Hak, 2013 (21)	Netherlands	2012–2013	Non-RCT	18	39.8	9/10	TTA	3 sessions, 1 day	Gait parameters
Hak, 2015 (22)	Netherlands	2013–2015	Non-RCT	10	range, 25–55	6/4	MS	12 sessions, 6 weeks, 2 times a week	Gait parameters
Sessoms, 2015 (23)	USA	2013	Non-RCT	24	31.9 ± 5.6	NR	TBI	12 sessions, 6 weeks, 2 times a week	Gait parameters
Kalron, 2016 (24)	Israel	2014–2015	Non-RCT	32	45	19/11	MS	12 sessions, 6 weeks, 2 times a week	Balance, mental health, and safety
Onakomaiya, 2017 (25)	USA	2010–2015	Non-RCT	214	mean, 35 (range, 20–63)	208/6	TBI	1 session	Balance, gait parameters, and mental health
He, 2018 (26)	China	2015–2016	RCT	90	range, 42–72	NR	stroke	80 sessions, 8 weeks, 10 times a week	Gait
Liang, 2019 (27)	China	2015–2016	RCT	24	8.3 ± 2.3	17/7	CP	36 sessions, 12 weeks, 3 times a week	Balance
Calabrò, 2020 (28)	Italy	2017–2018	Non-RCT	22	66 ± 4	18/4	PD	20 sessions, 5 weeks, 4 times a week	Balance, gait parameters, mental health, and safety
Rosen, 2021 (29)	USA	2010–2017	Non-RCT	97	34.2 ± 7.8	112/2	TBI	4 sessions	Balance, gait parameters, and mental health
Impellizzeri, 2022 (30)	Italy	2022	Non-RCT	15	62	9/6	PD	1 session	Cognition, mental health, and safety
Kane, 2022 (31)	USA	2013–2018	Non-RCT	75	51.5	50/25	MS	1 session	Mental health
Formica, 2023 (32)	Italy	2021	Single-arm	31	60 ± 5	16/15	PD	24 sessions, 8 weeks, 3 times a week	Balance, gait parameters, cognition, and mental health
Gerards, 2023 (33)	Netherlands	2019–2021	RCT	82	73	47/35	fallers	3 sessions, 3 weeks, 1 time a week	Balance, mental health, and safety
Impellizzeri, 2024 (34)	Italy	2022–2023	Non-RCT	40	65 ± 6	25/15	PD	24 sessions, 8 weeks, 3 times a week	Cognition, mental health, and safety

RCT, randomized controlled trial; M, male; F, female; TTA, traumatic transtibial amputation; MS, multiple sclerosis; TBI, traumatic brain injury; CP, cerebral palsy; PD, Parkinson’s disease; NR, not reported.

### CAREN scenarios training

As shown in Table 2, the different virtual reality scenarios and tasks used for training with the CAREN for the studies are provided. Virtual road environments and walking training tasks are the most common, with the majority using these tasks for walking training and target acquisition. Balance tasks, including the Balance Cubes and the Shark Hunt task, are also prevalent and are mostly used to enhance balance skills. Other studies use other types of tasks, including sea sailing, handball training, firefighting training, cross-slope training, and surface translation training, to enhance dynamic balance, weight shift, the capability to load the lower limbs, and walking coordination in patients.

**Table 2:** Virtual reality scenarios and tasks for CAREN training.

ID	Virtual reality scenario and task
Gates, 2012 (20)	Forest path gait training and gait balance task
Hak, 2013 (21)	Microbe and obstacle avoidance training
Hak, 2015 (22)	Virtual road scene, gait training, and target capture
Sessoms, 2015 (23)	Gait training and ship navigation task
Kalron, 2016 (24)	Virtual road scene, gait training, and target capture
Onakomaiya, 2017 (25)	Balance Balls, Balance Cubes, and Continuous Road
He, 2018 (26)	Forest walking, sea sailing, cyclic gait, and stepping training
Liang, 2019 (27)	Handball, sea sailing, fire-fighting drill, and gait training
Calabrò, 2020 (28)	Ship scene, balance, and obstacle avoidance training
Rosen, 2021 (29)	Balance Cubes and Shark Hunt task
Impellizzeri, 2022 (30)	Virtual road scene and gait training
Kane, 2022 (31)	Virtual road scene
Formica, 2023 (32)	Microbe scene, maze, and balance training
Gerards, 2023 (33)	Dynamic and static balance training scenes
Impellizzeri, 2024 (34)	Ship, step, and balance training scenes

### Quality assessment

The risk of bias for the included studies, presented in Supplementary Table S3, ranged from 5 to 8 on the Newcastle–Ottawa Scale (NOS). Among these, 9 studies were classified as having moderate risk (score: 5–7) and 6 studies as low risk (score: 7–8). The main areas of bias were “Selection of Controls” and “Comparability of Cases and Controls on the Basis of the Design or Analysis,” specifically concerning the selection of control groups and the comparison between the cases and the controls. Some studies also presented limitations for “Case Definition Adequate” and “Ascertainment of Exposure” that were responsible for the overall bias risk.

### Meta-analysis results

#### Balance

**Figure 2:**
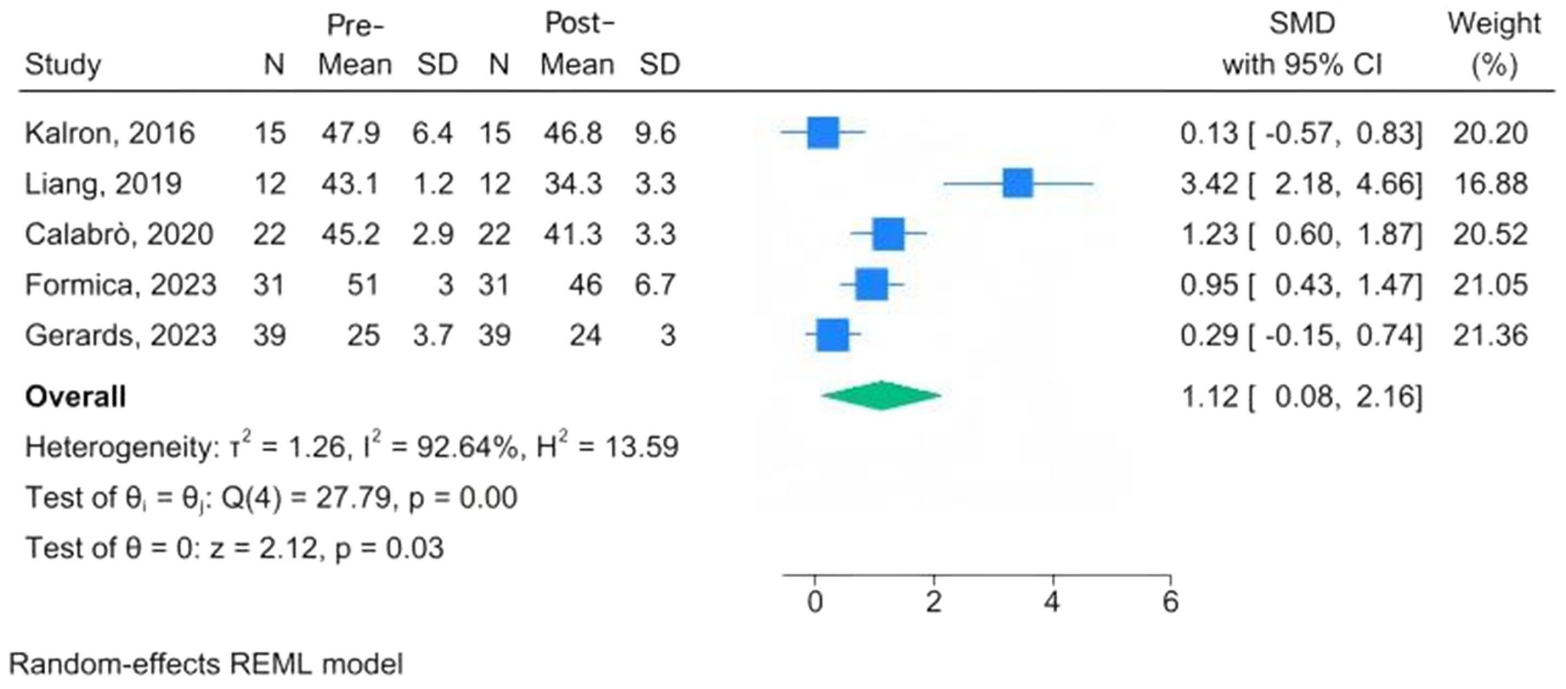
Forest plots of pre-post changes in balance following CAREN.

Assessment of balance function includes the Functional Reach Test, the time to perform the balance task, the tandem stance test, BBS, the Activities-Specific Balance Confidence Scale, the Mini-Balance Evaluation Systems Test, and the Sensory Organization Test. Five studies used standardized scales to quantify within-group changes following CAREN training in patients with MS, PD, fallers, and cerebral palsy (24, 27, 28, 32, 33). The pooled analysis suggested a trend toward improvement in balance following CAREN training (SMD = 1.12, 95% CI: 0.08 to 2.16; *p* = 0.03), although the very wide confidence interval and substantial heterogeneity (*I*^2^ = 92.6%) indicate considerable variability among the studies (Figure 2). Therefore, the balance-enhancing effect of CAREN should be interpreted with caution.

#### Cognitive functions

**Figure 3:**
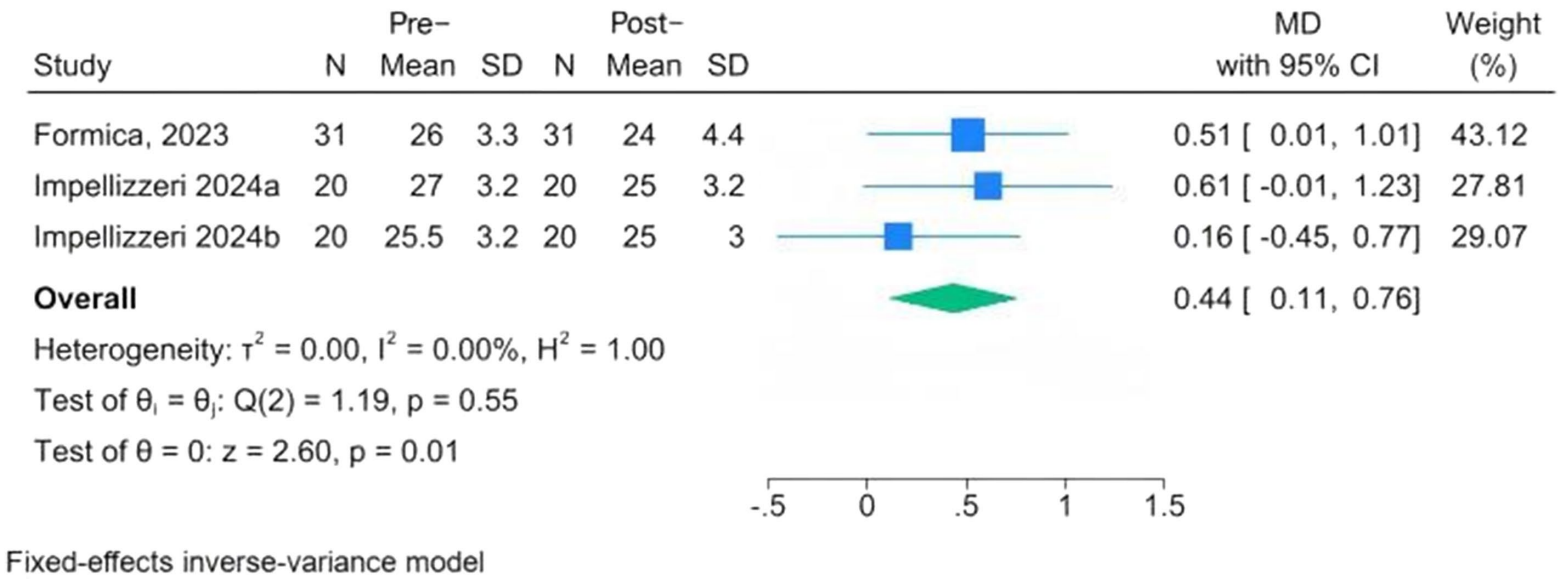
Forest plots of pre-post changes in cognitive function following CAREN.

Assessment for the cognitive functions includes the Stroop test, the Movement Disorder Society-Unified Parkinson’s Disease Rating Scale, and the MoCA. Only two studies (comprising three cohorts) provided quantitative MoCA data suitable for pooling (32, 34). The meta-analysis demonstrated a small but statistically significant within-group improvement in MoCA scores among PD patients after CAREN training (MD = 0.44, 95% CI: 0.11 to 0.76, *p* = 0.01). The *I*^2^ = 0.00% suggests that the findings from all the studies converged (Figure 3).

#### Mental health

**Figure 4:**
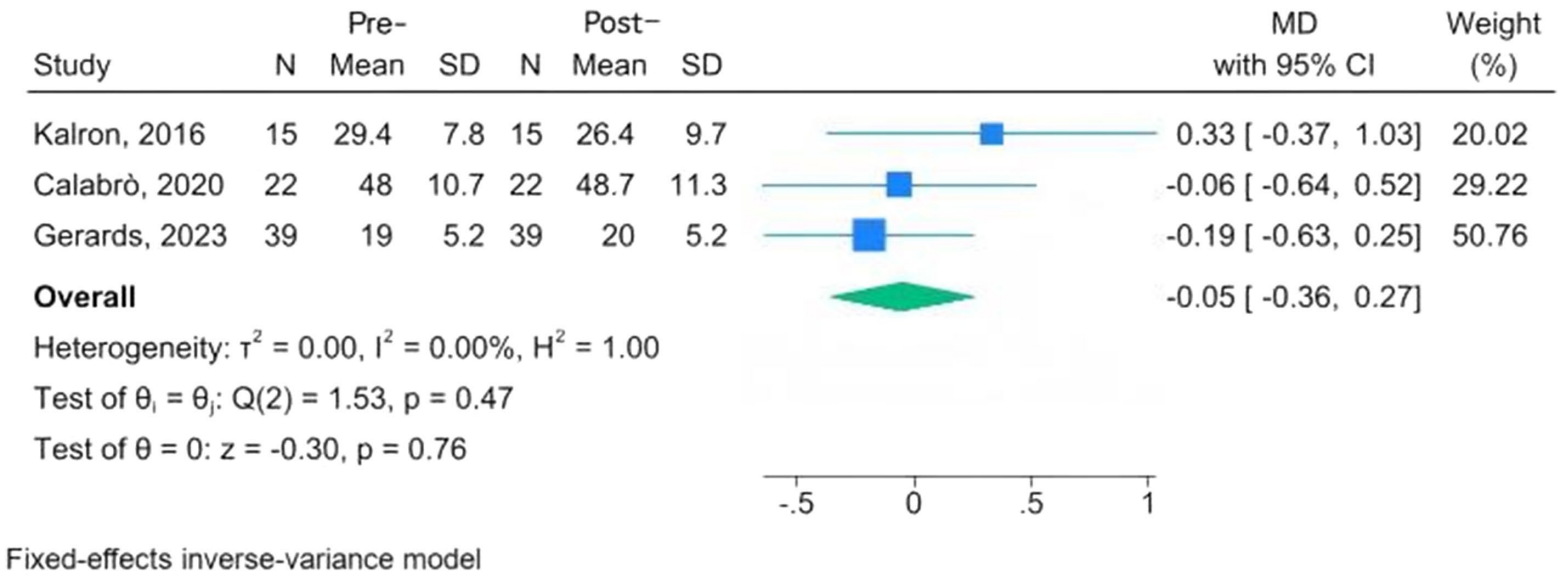
Forest plots of pre-post changes in fear of falling following CAREN.

**Figure 5:**
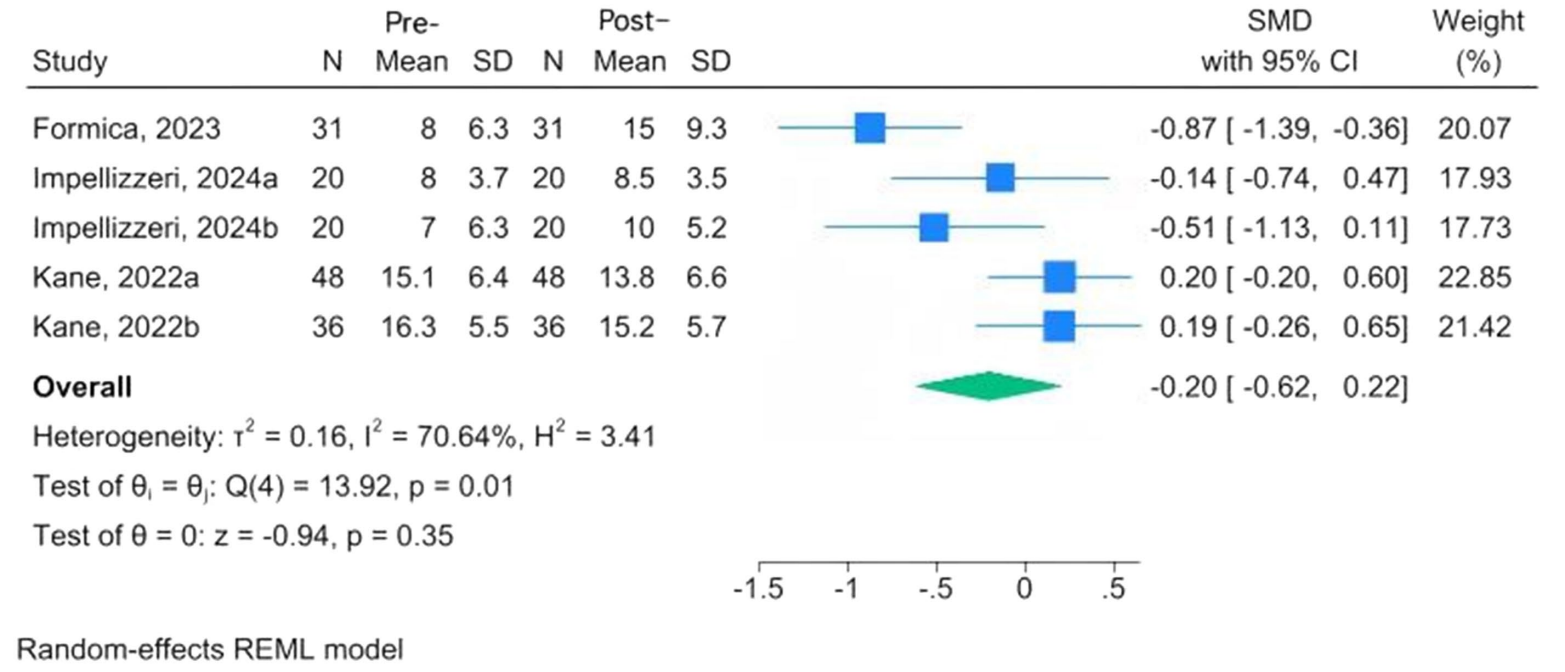
Forest plots of pre-post changes in depression following CAREN.

Mental health assessment tools are the FES-I, the Beck Inventory, Post-Traumatic Stress Disorder, the Hamilton Depression Rating Scale (HAMD), the Patient Health Questionnaire-9, and the Patient-Reported Outcomes Measurement Information System. Three studies, conducted among MS, PD, and fallers, used the FES-I; the overall results indicated a reduction in fear of falling following CAREN, but the change was not significant at the 95% confidence interval (MD = −0.05, 95% CI: −0.36 to 0.27; *p* = 0.76) (24, 28, 33). The *I*^2^ = 0.00% revealed that there was no significant heterogeneity (Figure 4). Three studies evaluated changes in depression scores before and after intervention among MS and PD patients undergoing CAREN (31, 33, 34). The overall evaluation showed a non-significant improvement following CAREN, with wide between-study heterogeneity (SMD = −0.20; 95% CI, −0.62 to 0.22; *p* = 0.35; *I*^2^ = 70.64%) (Figure 5).

#### Assessing the certainty of evidence

The meta-analysis found that CAREN training had potential benefits in balance (moderate certainty of evidence) and modest benefits in cognitive function (low certainty of evidence), with more limited evidence (moderate certainty) for fear of falling and depression outcomes, with continued very low certainty (Supplementary Table S4).

#### Adverse event

**Figure 6:**
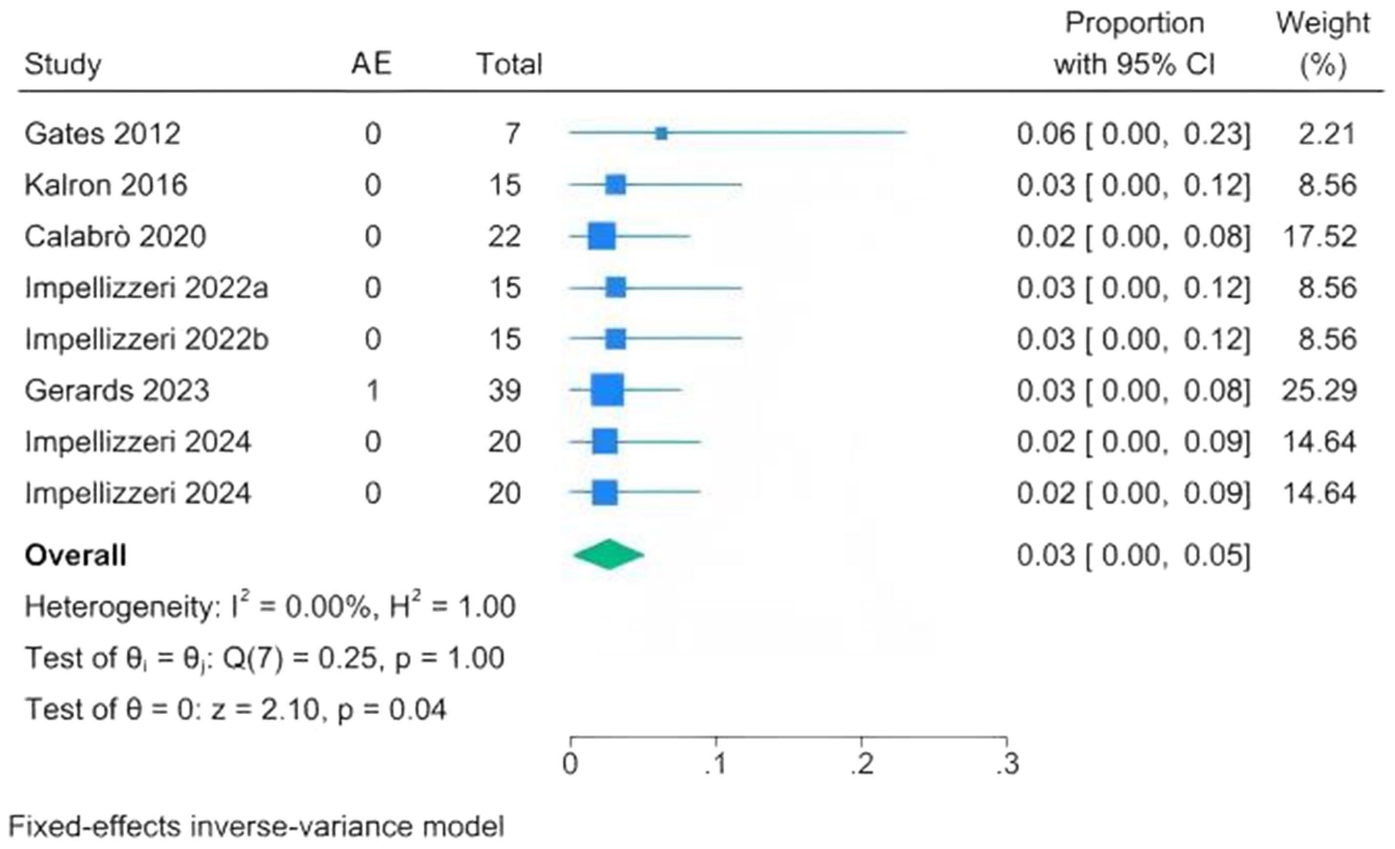
Forest plots of adverse event proportions following CAREN.

Due to limited reporting of adverse events in other intervention groups, the analysis focused on CAREN-related adverse events to evaluate safety (Supplementary Table S5). In the six studies (eight cohorts), only one mild adverse event, knee pain, was reported, which resolved within 2 days without intervention (20, 24, 28, 30, 33, 34). The incidence was 0.03 (95% CI 0.00–0.05; *I*^2^ = 0%), indicating that CAREN-based interventions are safe (Figure 6).

#### Publication bias

Funnel plots were visually inspected and did not suggest major asymmetry, although the small number of studies limits any firm conclusion (Supplementary Figure S1).

## Discussion

This analysis systematically assesses the rehabilitation benefit of CAREN treatment in patients. The included studies reported outcomes both before and after intervention, covering balance, gait, cognition, and mental health. We also observed that studies with musculoskeletal cohorts tended to place emphasis primarily on the outcome of gait, while those with neurological cohorts tended to include balance and mental health assessments more frequently, along with cognitive tests. It seems that training with the CAREN might be linked to possible improvements in balance; however, these results should be treated with caution due to differences in the patient populations (such as multiple sclerosis, stroke, and Parkinson’s disease) and variations in the intervention protocols (ranging from 3 to 36 sessions), which led to considerable heterogeneity in the results. Although the results suggest improvements in cognitive function, the difference (0.44) is unlikely to be clinically meaningful. Effects on mental health outcomes were limited and not statistically significant: fear of falling showed no clear change, and depressive symptoms exhibited only minimal improvement. AEs were rarely reported, supporting the overall safety of CAREN training.

The VR rehabilitation technology has been widely applied in neurological, musculoskeletal, and other diseases. Researchers found that VR-based interventions may improve balance in patients with knee pain and can also reduce pain intensity, particularly clear benefits for those with osteoarthritis (35). Investigators have also applied VR training to neurological populations, with results indicating that VR interventions can improve balance performance in patients with PD, MS, and stroke (36). In recent years, CAREN—an integrated immersive VR platform—has garnered increasing attention. Collins et al. reviewed its use in injured service members and reported improvements in gait, balance, and vestibular-related symptoms (37). A separate scoping review in neurological populations suggests that CAREN has potential utility for improving motor and cognitive skills, whereas findings regarding mood outcomes are mixed (38). We conducted the first meta-analysis focused exclusively on CAREN-based rehabilitation, building on prior narrative systematic reviews, and our findings further support these earlier reports.

CAREN applies immersive virtual reality to develop an ecologically valid, controllable rehabilitation environment that may support motor function recovery through mechanisms of neuroplasticity in patients with stroke and Parkinson’s disease (39, 40). With the combination of extensive visual–vestibular stimulations and synchronized movement platforms and real-time kinematic feedback, the system recruits motor circuits, facilitates motor planning, and enhances gait symmetry, walking speed, and postural stability (41). Interactive, task-specific scripts provide progressive challenge, perturbation training, and dual-task training that build balance confidence without exacerbating fear of falling. In musculoskeletal diseases involving fractures and arthritis, the low-load, high-repetition training decreases joint load with functional specificity, promoting a safer return to activity (42, 43). Therapists can individualize programs, monitor objective measures, and provide motivational support through interactive VR tasks that have been associated with better adherence and potentially improved outcomes.

We noted that CAREN training correlated with significant balance improvements, perhaps because tasks are aimed at postural control-oriented functions (e.g., weight shift, perturbation exposure, and stance stabilization) (44). Such paradigms may facilitate sensory reweighting (vestibular-proprioceptive-visual integration) and error-based learning through augmented feedback, while repeated anticipatory and reactive postural adjustments practiced might enlist plasticity at cortico-cerebello-basal ganglia circuits active with postural control (45, 46). Cognitive benefits, by contrast, were modest, likely due to the limited transfer from the embedded dual task/visuospatial requirements and an insufficient cognitive dose or target specificity (e.g., too few modules directly training memory or executive functions) (34, 47). Mental health benefits were not observed, in keeping with effects that typically require longer exposure and explicit psychological components (e.g., graded exposure, CBT-informed components, affect regulation, and motivational enhancement); All included studies were motor-oriented (31, 48). Furthermore, there might be significant variations in the “dose” of intervention (number, duration, and frequency), which can also impact the extent to which improvements are realized.

Several limitations exist for this study. First, the small number of eligible studies with quantitative data significantly limited statistical robustness and the power to detect meaningful effects, making it difficult to draw firm conclusions; moreover, the majority of studies focused on neurological disorders. Second, the wide variation in CAREN intervention protocols—such as differences in virtual reality environments, training frequency, and dosage—contributed to considerable heterogeneity, affecting the consistency and generalizability of the findings. Third, regular long-term follow-up is not feasible and consequently diminishes the power to draw conclusions regarding the sustainability and safety of the effects of CAREN. Fourth, the study compared rehabilitation outcomes before and after the intervention but could not control for confounding variables or biases, such as regression to the mean or natural improvement, which may have influenced the true intervention effect. Finally, comprehensive inclusion enhances external validity, but the pathophysiological heterogeneity and variable/measured reporting of the outcome inflate heterogeneity and diminish effect estimates, study comparability, and generalizability to particular groups.

## Conclusion

Although CAREN training appears to support improvements in balance and cognition, these findings should be interpreted with caution, and evidence regarding its effects on mental health remains limited. Future trials should standardize the dosing and task content, incorporate extended follow-up periods, and consider incorporating cognitive and psychological targeted elements to further increase the rehabilitative advantage afforded by the CAREN.

## Data Availability

The original contributions presented in the study are included in the article/Supplementary material, further inquiries can be directed to the corresponding author.
