# Supplementary material for: Effects of the computer-assisted rehabilitation environment on patient rehabilitation: a systematic review and meta-analysis
Source: Front Med (Lausanne). 2025 Dec 5;12:1733273. doi: 10.3389/fmed.2025.1733273 (PMC12714871; doi:10.3389/fmed.2025.1733273)

**Supplementary files**

**List of Supplementary Files**

Table S1. Literature search strategy for included studies

Table S2. Summary of comparator interventions and control groups in CAREN-based studies

Table S3. Risk of bias summary using the Newcastle-Ottawa Scale

Table S4. GRADE assessment of evidence certainty for each outcome

Table S5. Incidence of adverse events in included studies

Figure S1. Funnel plots for CAREN effects across outcomes. (A), balance; (B), cognitive function; (C), fear of falling; (D), depression; (E), adverse events.

**Table S1. Literature search strategy for included studies <2025-11-09>**

| **Database** | **#** | **Run date:** | **Searches** | **Results** |
| --- | --- | --- | --- | --- |
| **Pubmed** | 1 | **2025-11-09** | "Computer-Assisted Rehabilitation Environment"[Title/Abstract] OR "CAREN"[Title/Abstract] OR "virtual reality"[MeSH Terms] OR "virtual reality-based rehabilitation"[Title/Abstract] OR "computer-assisted rehabilitation"[Title/Abstract] OR "robot-assisted rehabilitation"[Title/Abstract] OR "immersive rehabilitation"[Title/Abstract] | 10,001 |
|  | 2 |  | "Rehabilitation"[MeSH Terms] OR "neurorehabilitation"[Title/Abstract] OR "motor recovery"[Title/Abstract] OR "balance"[Title/Abstract] OR "gait"[Title/Abstract] OR "cognitive function"[Title/Abstract] OR "Cognition"[MeSH Terms] | 1,024,567 |
|  | 6 |  | 1 AND 2 | 2166 |
|  | 7 |  | NOT animals | 2139 |
| **Embase** |  | **2025-11-09** |  |  |
|  | 1 |  | 'computer assisted rehabilitation environment':ti,ab OR 'CAREN':ti,ab OR 'virtual reality'/exp OR 'virtual reality':ti,ab OR 'immersive':ti,ab OR 'virtual reality-based rehabilitation':ti,ab OR 'computer assisted rehabilitation':ti,ab OR 'robot assisted rehabilitation':ti,ab OR 'immersive rehabilitation':ti,ab | 13,209 |
|  | 2 |  | 'rehabilitation'/exp OR 'neurorehabilitation':ti,ab OR 'motor function'/exp OR 'balance'/exp OR 'gait'/ti,ab OR 'cognition'/ti,ab OR 'depression'/ti,ab OR 'anxiety'/ti,ab | 493,318 |
|  | 6 |  | 1 AND 2 | 2246 |
|  | 7 |  | NOT animals | 1330 |
| **Web of Science** | **#** | **2025-11-09** |  |  |
|  | 1 |  | TS=("Computer-Assisted Rehabilitation Environment" OR "CAREN" OR "computer assisted rehabilitation" OR "robot assisted rehabilitation" OR "immersive rehabilitation") | 848 |
|  | 2 |  | TS=("rehabilitation" OR "neurorehabilitation" OR "motor function" OR "balance" OR "gait" OR "cognitive function" OR "depression" OR "anxiety" OR "fear of falling") | 2,218,161 |
|  | 6 |  | 1 AND 2 | 668 |
|  | 7 |  | NOT animals | 667 |
| **Cochrane Library** | **#** | **2025-11-09** |  |  |
|  | 1 |  | ("Computer-Assisted Rehabilitation Environment" OR "CAREN" OR "computer assisted rehabilitation" OR "robot assisted rehabilitation" OR "immersive rehabilitation") in Title Abstract Keyword | 178 |
|  | 2 |  | TS=("rehabilitation" OR "neurorehabilitation" OR "motor function" OR "balance" OR "gait" OR "cognitive function" OR "depression" OR "anxiety" OR "fear of falling") | 315,506 |
|  | 6 |  | 1 AND 2 | 160 |
|  | 7 |  | NOT animals | 160 |
| **CNKI** | **#** | **2025-11-09** |  |  |
|  | 1 |  | ("Computer-Assisted Rehabilitation Environment" OR "CAREN" OR "computer assisted rehabilitation" OR "robot assisted rehabilitation" OR "immersive rehabilitation") in Title+keywords+abstract | 62 |
|  | 2 |  | TS=("rehabilitation" OR "neurorehabilitation" OR "motor function" OR "balance" OR "gait" OR "cognitive function" OR "depression" OR "anxiety" OR "fear of falling") in Title+keywords+abstract | 1,532 |
|  | 6 |  | 1 AND 2 | 137 |
|  | 7 |  | NOT animals | 137 |
| **Totle: 5** |  | **2025-11-09** |  | **4433** |

**Table S2. Summary of comparator interventions and control groups in CAREN-based studies**

| **Study** | **CAREN Group** | **Control Group** |
| --- | --- | --- |
| Gates (2012) | CAREN | No intervention |
| Hak (2013) | CAREN | No intervention |
| Hak (2015) | CAREN | No intervention |
| Sessoms (2015) | CAREN | Traditional physical therapy |
| Kalron (2016) | CAREN | Conventional balance exercise program |
| Onakomaiya (2017) | CAREN (TBI+ PTSD) | CAREN (only TBI) |
| He (2018) | CAREN+acupuncture | Acupuncture+conventional therapy |
| Liang (2019) | CAREN | Traditional training + routine rehabilitation |
| Calabrò (2020) | CAREN | Traditional training |
| Rosen (2021) | CAREN (TBI+ PTSD) | CAREN (only TBI) |
| Impellizzeri (2022) | CAREN | CAREN (healthy control) |
| Kane (2022) | CAREN (≥ 5 sessions) | CAREN (1–4 sessions) |
| Formica (2023) | CAREN | No control group |
| Gerards (2023) | CAREN | Traditional physical therapy |
| Impellizzeri (2024) | CAREN + Music | CAREN |

**Table S3. Risk of bias summary using the Newcastle-Ottawa Scale**

| ID | Is the Case Definition Adequate? | Representativeness of the Cases | Selection of Controls | Definition of Controls | Comparability of Cases and Controls on the Basis of the Design or Analysis | Ascertainment of exposure | Same method of ascertainment for cases and controls | Non-Response rate | Total scores |
| --- | --- | --- | --- | --- | --- | --- | --- | --- | --- |
| Gates, 2012 | ★ | ☆ | ★ | ★ | ★★ | ★ | ★ | ★ | 8 |
| Hak, 2013 | ★ | ☆ | ★ | ★ | ★★ | ★ | ★ | ☆ | 7 |
| Hak, 2015 | ★ | ☆ | ☆ | ☆ | ★★ | ★ | ★ | ★ | 6 |
| Sessoms, 2015 | ★ | ☆ | ☆ | ☆ | ★☆ | ★ | ★ | ★ | 5 |
| Kalron, 2016 | ★ | ☆ | ★ | ★ | ★★ | ★ | ★ | ★ | 8 |
| Onakomaiya, 2017 | ★ | ★ | ☆ | ☆ | ★☆ | ★ | ★ | ☆ | 6 |
| He, 2018 | ★ | ☆ | ☆ | ★ | ★☆ | ★ | ★ | ★ | 6 |
| Liang, 2019 | ★ | ★ | ☆ | ☆ | ★☆ | ★ | ★ | ★ | 6 |
| Calabrò, 2020 | ★ | ☆ | ☆ | ☆ | ★☆ | ★ | ★ | ★ | 5 |
| Rosen, 2021 | ★ | ☆ | ★ | ☆ | ★☆ | ★ | ★ | ☆ | 5 |
| Impellizzeri, 2022 | ★ | ☆ | ★ | ★ | ★☆ | ★ | ★ | ☆ | 6 |
| Kane, 2022 | ★ | ☆ | ☆ | ☆ | ★☆ | ★ | ★ | ★ | 5 |
| Formica, 2023 | ★ | ★ | ☆ | ☆ | ★★ | ★ | ★ | ★ | 7 |
| Gerards, 2023 | ★ | ★ | ★ | ★ | ★★ | ★ | ★ | ☆ | 8 |
| Impellizzeri, 2024 | ★ | ☆ | ★ | ★ | ★☆ | ★ | ★ | ★ | 7 |

**Table S4. GRADE assessment of evidence certainty for each outcome**

| **Outcome** | **Study design** | **Risk of bias** | **Inconsistency** | **Indirectness** | **Imprecision** | **Publication bias** | **Final certainty** |
| --- | --- | --- | --- | --- | --- | --- | --- |
| Balance | 3 RCTs, 2 NRCTs | Moderate | Serious | Not serious | Serious | Not serious | Moderate |
| Cognitive function | All NRCTs | Serious | Not serious | Not serious | Serious | Not serious | Low |
| Fear of falling | 2 RCT, 1non-RCT | Moderate | Not serious | Not serious | Serious | Not serious | Moderate |
| Depression | All NRCTs | Serious | Serious | Serious | Serious | Not serious | Very low |

**Table S5. I Incidence of adverse events in included studies**

| **Study** | **Intervention group** | | | **Control group** | | |
| --- | --- | --- | --- | --- | --- | --- |
|  | **Report adverse events** | **n** | **AE** | **Report adverse events** | **n** | **AE** |
| Gates 2012 | Yes | 7 | 0 | Yes | 27 | 0 |
| Hak 2013 | No | 9 | - | No | 9 | - |
| Hak 2015 | No | 10 | - | No | 10 | - |
| Sessoms 2015 | No | 12 | - | No | 12 | - |
| Kalron 2016 | Yes | 15 | 0 | Yes | 15 | 0 |
| Onkomaiya 2017 | No | 148 | - | No | 66 | - |
| He 2018 | No | 30 | - | No | 60 | - |
| Liang 2019 | No | 12 | - | No | 12 | - |
| Calabrò 2020 | Yes | 22 | 0 | No | 22 | - |
| Rosen 2021 | No | 59 | - | No | 38 | - |
| Kane 2022 | No | 34 | - | No | 41 | - |
| Impellizzeri 2022* | Yes | 15 | 0 | Yes | 15 | 0 |
| Gerards 2023 | Yes | 39 | 1 | No | 43 | - |
| Formica 2023 | No | 31 | - | - | - | - |
| Impellizzeri 2024* | Yes | 20 | 0 | Yes | 20 | 0 |
| *, Both groups received CAREN intervention, differing in task design or participant characteristics. | | | | | | |

Figure S1. Funnel plots for CAREN effects across outcomes. (A), balance; (B), cognitive function; (C), fear of falling; (D), depression; (E), adverse events.


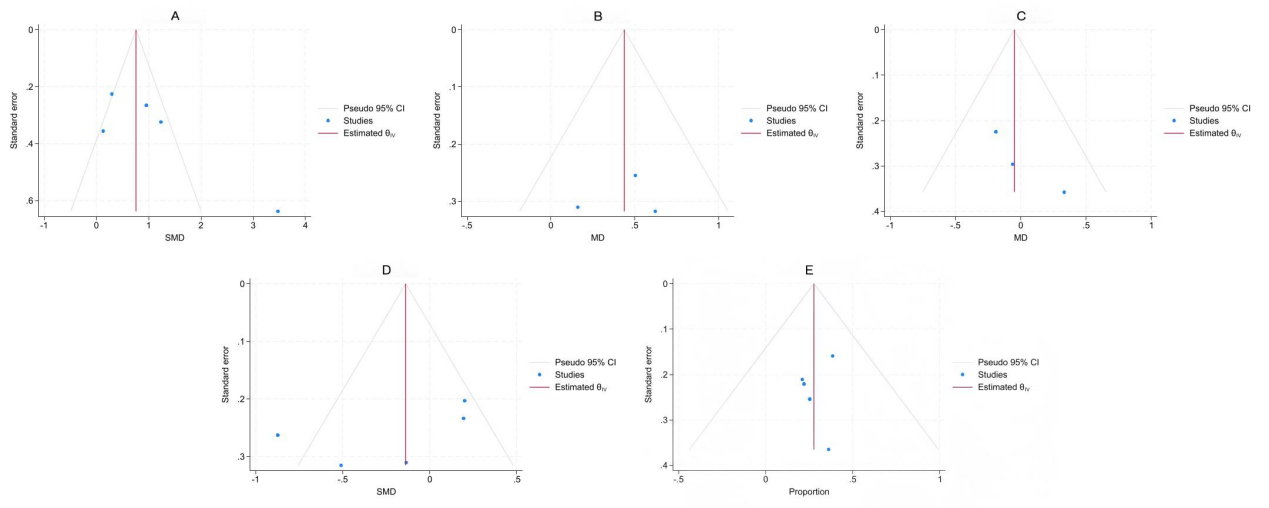

Supplement: Supplementary file 1 [file Data_Sheet_1.docx]
